# Characterization of *Mycobacterium Abscessus* Subtypes in Shanghai of China: Drug Sensitivity and Bacterial Epidemicity as well as Clinical Manifestations

**DOI:** 10.1097/MD.0000000000002338

**Published:** 2016-01-22

**Authors:** Liulin Luo, Bing Li, Haiqing Chu, Dongdong Huang, Zhemin Zhang, Jingbo Zhang, Tao Gui, Liyun Xu, Lan Zhao, Xiwen Sun, Heping Xiao

**Affiliations:** From the Department of Clinical Laboratory Medicine, Shanghai Pulmonary Hospital, Tongji University School of Medicine (LL); Department of Respiratory Medicine, Shanghai Pulmonary Hospital, Tongji University School of Medicine (BL, HC, ZZ, JZ, TG, LX, LZ); Department of Clinical Medicine, Shanghai Pulmonary Hospital, Tongji University School of Medicine (DH); Department of Radiology, Shanghai Pulmonary Hospital, Tongji University School of Medicine (XS); and Department of Tuberculosis, Shanghai Pulmonary Hospital, Tongji University School of Medicine, Shanghai, China (HX).

## Abstract

The aim of the study was to investigate the epidemic characteristics of *Mycobacterium abscessus* in Shanghai.

Fifty-five strains from 55 *M. abscessus* pulmonary disease patients were isolated. Drug sensitivity was measured by a broth microdilution method. Subtypes of *M. abscessus* were identified by DNA sequencing. Multilocus sequence typing (MLST), mining spanning tree (MST), and pulsed-field gel electrophoresis (PFGE) were used to analyze sequence types (ST) and clonal complexes (CC). Clinical manifestations were assessed by CT imaging.

We identified 42 A isolates, 11 M, and 2 B-subtypes. A and M were highly sensitive to tigecycline and amikacin (97.6–100%). The A-type easily developed drug resistance against clarithromycin. Both types were highly resistance to sulfonamides, moxifloxacin, doxycycline, imipenem, and tobramycin. MLST analysis identified 41 STs including 32 new STs. The MST algorithm distributed 55 isolates into 12 separate CC. The PFGE analysis exhibited 53 distinct restriction patterns and the M-type was closely clustered according to their ST and CC numbers. CT imaging showed that tree-in-bud and patch shadow were commonly observed in M-type, whereas pulmonary cavities were often found in A-type infection patients (*P* < 0.001).

ST1 in A and ST23 in M-type were the main epidemic strains in Shanghai. The M-type appeared to be prone to epidemic nosocomial transmission.

## INTRODUCTION

Nontuberculous mycobacteria (NTM) have an extensively wide pathological spectrum including NTM pulmonary diseases, lymphadenopathy, and skin and soft tissue infections.^1^ Because of their ubiquitous distribution, NTM can cause nosocomial infections and epidemic outbreaks.^2–4^ In recent years, the incident of NTM infectious diseases has significantly increased due to the ascending NTM infection rate, especially among immunocompromised patients, and an increase in invasive surgical operations. The development of molecular diagnostic technology has shown that the diseases caused by NTM cannot be neglected in the clinic.^5^

NTM is divided into 2 groups, namely rapidly growing mycobacteria (RGM) and slowly growing mycobacteria. RGM, for example, can cause an extensive pulmonary infection. In Japan and America, ∼5% of pulmonary infections are caused by RGM,^6^ and ∼65% to 80% of RGM infections are due to *Mycobacterium abscessus*.^6–11^ An American group showed that *M. abscessus* was responsible for >80% of pulmonary infections caused by rapidly growing NTM.^6^ A Korean study demonstrated that *M. abscessus* ranked only second to *Mycobacterium avium*, accounting for 12% of 17,915 NTM strains clinically isolated.^9^ The Brazilian government reported the identification of *M. abscessus* infection in ∼2000 patients at surgical sites, and this agent once caused a state of emergency after an epidemic outbreak from 2004 to 2008.^12^ Besides pulmonary disease, *M. abscessus* can also cause lesions of lymph nodes, skin soft tissues and bone joints, and general pathological infections.^11^ The bacteria easily elicits outbreaks of infection in postsurgical wounds,^8^ and a disseminated infection in cystic fibrosis patients after transplantation.^11^ Currently, treatment of *M. abscessus* pulmonary disease relies on broad-spectrum antibiotics combined with others. However, an occurrence of drug resistance and natural resistance to antituberculosis drugs often makes the treatment ineffective. Although antibiotics such as amikacin, cefoxitin, or imipenem are known to be effective against *M. abscessus,* there is a high occurrence of drug resistance and also intolerable gastrointestinal toxicity. Thus, the prognosis of *M. abscessus* pulmonary disease is presently not promising.^7^

According to the differences in DNA sequences of heat shock protein 65 (hsp65), RNA polymerase β (rpoB), and erythromycin ribosomal methylase 41, known as erm(41), and the differences of drug sensitivity, *M. abscessus* is divided into 3 subtypes: *M. abscessus* (A-type), *M. massiliense* (M-type), and *M. bolletii* (B-type).^13–15^ Some studies have shown differences between subtypes of *M. abscessus* in terms of their biological characteristics, antibiotic sensitivity, and clinical features (efficacy and prognosis are unpredictable),^16–18^ indicating a complex molecular identification, molecular typing, and drug sensitivity profile.

Subtype analysis is a powerful technique for discovering and controlling an epidemic outbreak, and also for treating patients with *M. abscessus* infections. However, in the Shanghai district of China, molecular typing and analysis of the phylogenetic relationships of *M. abscessus* strains, as well as the corresponding clinical material analysis, have not been systemically carried out.

The aim of the present study was to investigate the epidemic characteristics of *M. abscessus* in the Shanghai district. We analyzed the antibiotic sensitivities of the bacteria subtypes, evaluated the evolution and epidemic dissemination of the strains with molecular typing, and identified the clinical features and characteristics of the subtypes, in order to provide reference evidence for the diagnosis and treatment of *M. abscessus* infection in the Shanghai district.

## MATERIALS AND METHODS

### Patient Material, Bacteria Culturing and Identification

Materials from 55 patients who suffered from *M. abscessus* pulmonary disease were retrospectively analyzed. Patients underwent the initial diagnosis and treatment at the Shanghai Pulmonary Hospital Affiliated to Tongji University from January 2013 to December 2014, and the diagnostic criteria were in accordance with the American Thoracic Association Guidelines.^16^ All patients were given a CT scan and sputum, lavage fluid, pleural effusion, and biopsy samples were collected to aid the initial diagnosis. *Mycobacterium abscessus* infection was confirmed by bacterial culture and strain identification, and all the specimens were preserved at −80°C for further investigations. The study was approved by the Ethical Committee of Tongji University and by the Shanghai Pulmonary Hospital. All the participants signed informed consent forms before being enrolled.

A total of 55 microbiological specimens were inoculated on the slope of acid Lowenstein–Jensen (L–J) medium after alkali treatment with 4% NaOH. Cultivated bacterial colonies were acid-fast stained with auramine O (1 mg/mL) and then examined microscopically to identify acid-fast bacteria colonies. To select further NTM, positive colonies were inoculated and cultured in L-J medium with P-nitrobenzoic acid (0.5 mg/mL) and 2-thiophenecarboxylic acid hydrazide (5 μg/mL) at 37°C for 1 to 2 weeks. Bacteria isolates that rapid grew in this medium were selected for molecular typing analysis using PCR. To prepare DNA, bacteria were split with lysozyme (1 mg/mL) and digested with proteinase K (1 mg/mL) followed by phenol/chloroform exaction. First, the rpoB gene was amplified by PCR and the DNA sequences were determined. To confirm the *M. abscessus* complexes, 754 bp of the DNA segment was subjected to BLAST analysis. Second, the erm(41) gene was amplified and the DNA sequence was analyzed to identify M (*M. massiliense*), A (*M. abscessus*), and B (*M. bolletii*) subspecies. Finally the PRA-hsp65 gene was compared to an online reference (http://app.chuv.ch/prasite/index.html), when the A- and B-types were confirmed. Sequences and the length of primers are shown in Table 1. The methods were carried out in “accordance” with the approved guidelines.

**TABLE 1:**
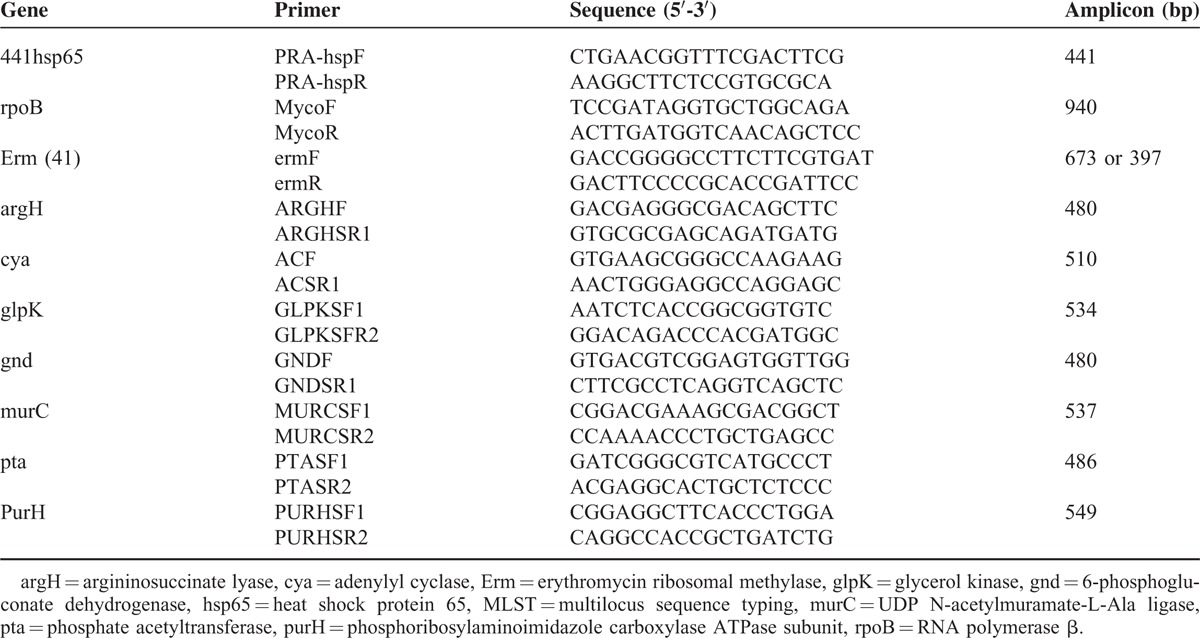
DNA Sequences of Primers Used for Multilocus Sequence Typing (MLST) and Subtyping

### Drug Sensitivity Assay

A broth micro-dilution method was used for the drug sensitivity assay. Bacteria were added to Middlebrook 7H9 medium containing glass beads (3 mm) for enrichment. Then an appropriate quantity of bacteria were suspended and diluted in a 3 mL saline solution to produce a turbidity of 0.5 McFarland (MFC). Each 50 μL suspension was inoculated in 11 mL of cation-adjusted Mueller-Hinton medium and then dispersed into 100 μL medium containing different concentrations of antibiotics in 96-well microtiter plates producing a final bacteria concentration of 10^5^ CFU/mL. After culturing for 72 h at 37°C, growth was assessed and drug sensitivity was calculated according to CLSI-M24-A2 criteria.^19^ The antibiotics tested were sulfonamides, moxifloxacin, cefoxitin, amikacin, doxycycline, tigecycline, clarithromycin, linezolid, imipenem, and tobramycin (TREK Diagnostic Systems, USA).

### Phylogenetic Analysis of Strains by Multilocus Sequence Typing (MLST) and Mining Spanning Tree (MST)

The following 7 housekeeping genes were used for MLST sequencing analysis: argininosuccinate lyase (argH); adenylyl cyclase (cya); glycerol kinase (glpK); 6-phosphogluconate dehydrogenase (gnd); UDP N-acetylmuramate-L-Ala ligase (murC); phosphate acetyltransferase (pta); (phosphoribosylaminoimidazole carboxylase ATPase subunit (purH). The DNA sequence of primer pairs for each gene is shown in Table 1. The sequences were modified according to references in the MLST website (http://www.pasteur.fr/recherche/genopole/PF8/mlst/primersabscessus.html Consensus sequences were established using the Bioedit software program ver. 7.1.3.0 and were compared and analyzed according to references in the MLST (http://www.pasteur.fr/cgi-bin/genopole/PF8/mlstdbnet.pl?page=oneseq&file=Mabscessus_profiles.xml) website. Acquired information of allele combinations was submitted to the allele distribution inquiry website (http://www.pasteur.fr/cgi-bin/genopole/PF8/mlstdbnet.pl?page=profile–query&file=Mabscessus_profiles.xml) and sequence type (ST) numbers were obtained. Information of newly found alleles was submitted to the MLST database and alleles were given new ST numbers.

The neighbor-joining (NJ) method was used to analyze MLST sequencing results with the bootstrap confidence interval set at 2000. Each sample contained information about a 3756 bp DNA sequence. The maximum likelihood method was used to calculate the phylogenetic relationship, and Mega 6.06 software was used for the analysis. Established strains ATCC19977, CIP18297, and CIP108541 served as the reference control strains for A-, M-, and B-types, respectively.

MST was used for evolution family analysis. STs were clustered according to the MLST sequencing results in order to create clonal complexes (CC). Each CC was composed of 2 or more STs, and each ST contained 6 of the 7 housekeeping genes. If 3 or more of these genes showed a difference, the ST was regarded as a singleton. Therefore, in the same CC, the ST ancestor could be located in the center of this CC.

### Analysis of Epidemic Strains by Pulsed-Field Gel Electrophoresis (PFGE)

All bacteria isolates were subjected to PFGE as previously described.^6,11^ Logarithmically growing bacteria were collected, washed with TE buffer (50 mM Tris: 50 mM EDTA) and re-suspended in EC buffer (50 mM Tris, 50 mM EDTA, pH 8.0 + 1% sarcosyl, and 1 mg/mL lysozyme). After adding lysozyme at a final concentration of 1 mg/mL, the suspension was incubated at 55°C for 2 h. Then the suspension was rapidly mixed with an equal volume of EC buffer containing 2% low-temperature-melting agarose and cast into plug molds. The plugs were allowed to solidify at 4°C, and placed in EC buffer containing lysozyme at a final concentration of 1 mg/mL, followed by shaking in an incubator overnight. Lysozyme containing EC buffer was replaced with cell lysis buffer (CLB) (50 mM Tris:50 mM EDTA, pH 8.0 + 1% Sarcosyl) and protease K was added at a final concentration of 1 mg/mL, and the plugs were incubated in a shaking bath at 55°C for 1 h. Then, the plugs were washed with TE buffer 3 times by shaking at 55°C for 15 min and were then placed in TE buffer containing 30 U Ase I restriction enzyme (New England Biolabs, UK) followed by incubation at 37°C for 3 h. After enzyme digestion, the plugs were loaded onto CHEF Mapper XA electrophoresis apparatus (Bio-Rad, USA) with 1% agarose and subjected to electrophoresis with a molecular standard marker. To obtain the PFGE gel images, the gels were stained with Gel red (1 μg/mL) (Biotium, USA) and placed in the imaging apparatus (Bio-Rad GelDoc XR+). The images were analyzed by BioNumericsver. 5.1 (Applied Maths, Sint-Martens-Latem, Belgium). The Dice's coefficient correlation was used for fingerprint chromatography analysis with a tolerance index of 1%. Bacteria genotype grouping and classification as well as genotype similarity were attained utilizing the calculation of the unweighted pair group method with arithmetic mean (UPGMA).

### CT Scanning

All CT images were obtained from the Radiology Department of Shanghai Pulmonary Hospital. CT scanning was performed within 3 months from the starting date of the acid-fast bacillus (AFB) smear test. Multislice spiral CT (Philips Brilliance 64) was used for routine CT scanning with 5 mm thick images at 5 mm intervals. High-resolution computed tomography (HRCT) images were obtained by scanning with a 1 mm thickness at 10 mm intervals. The scanning range was from the lung apex downward extending to the costophrenic angle under maximum inhalation. CT images were analyzed and interpreted by 4 CT imaging experts and 2 pulmonary disease experts who were blinded for the microbiological test results. The final interpretation was established by the consensus made by 3 experts. CT manifestations were evaluated according to the descriptions shown in Table 6.

**TABLE 6:**
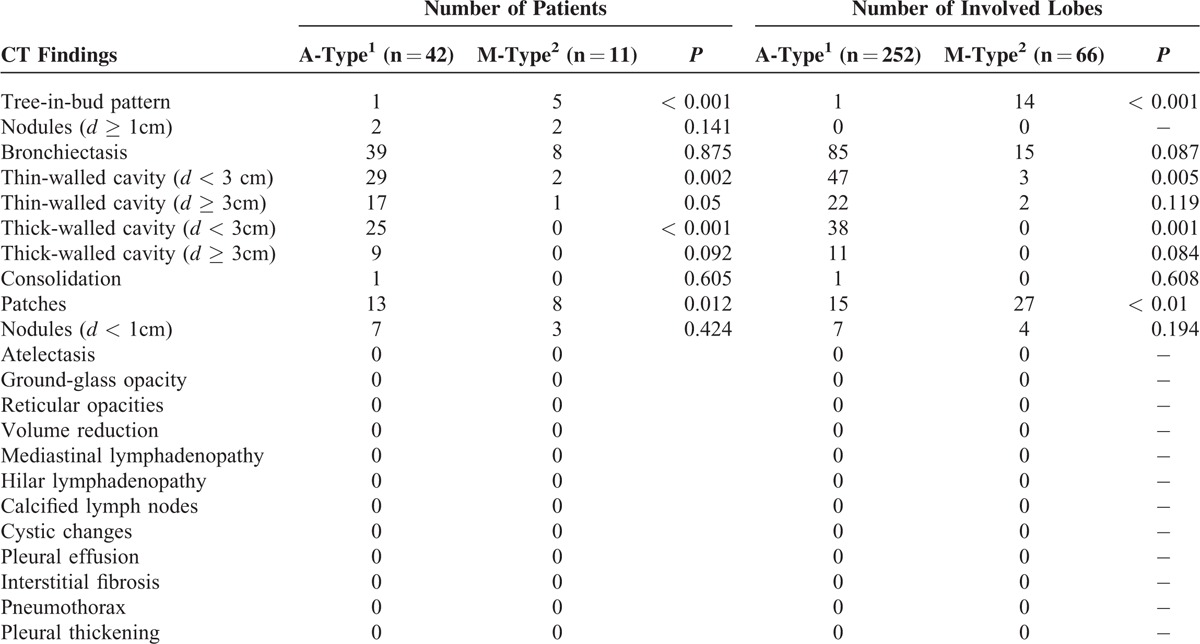
Comparative Chest CT Findings of Patients With *M. abscessus* (A-type) and *M. massiliense* (M-Type)

### Statistical Analysis

SPSS ver. 17.0 software (IBM SPSS Statistics for Windows, Version 17.0 Armonk, NY: IBM Corp) was used for statistical analysis. A chi-square test or Fisher's exact test was used for the comparison of categorical variables. The independent nonpaired *t* test was used for the comparison of continuous variables. *P* < 0.05 was regarded as statistically significant.

## RESULTS

### Subtype Identification of Bacteria Strains

Fifty-five bacteria isolates were identified as *M. abscessus* complexes by MLST sequencing and BLAST comparative analysis of the rpoB gene. Among these, 11 strains were identified as M-type based on the result that their amplified fragment size of the erm(41) gene was 397 bp lacking 276 bp clarithromycin-resistance regions. The fragment size of the remaining strains was 673 bp, suggesting they were A- or B-types. To further identify A- and B-types, analysis of the restriction enzyme sites of the amplified fragments of the PRA-hsp65gene (441 bp) was performed. Forty-two strains were identified as A-type, showing restriction patterns of BstE II 235 / 210 and HaeIII145 / 70 / 60 / 55, whereas 2 strains were identified as B-type showing those of BstE II 235 / 210 and HaeIII200 / 70 / 60 / 50.

### Comparison of Drug Sensitivity Between Subtypes

A- and M-type strains were tested for drug sensitivity; however, the B-type was excluded because of its minute number of isolates. The results demonstrate that both strains were highly sensitive to intravenous antibiotics, such as tigecycline and amikacin, exhibiting sensitivity rates of 100% and 97.6%, respectively, against A-type and 100% and 100%, respectively, against the M-type. However, differences in the sensitivity between these 2 subtypes were not statistically significant (*P* = 1.0) (Table 2). Both subtypes showed moderate sensitivity to cefoxitin, clarithromycin, and linezolid, with the sensitivity ranging from 71.4% to 90.9%. Drug resistance against clarithromycin was easily induced in the A-type strain; in contrast, almost no induction was observed in the M-type. Both types showed a relatively high resistance with no statistically significance differences against sulfonamides, moxifloxacin, doxycycline, imipenem, or tobramycin.

**TABLE 2:**
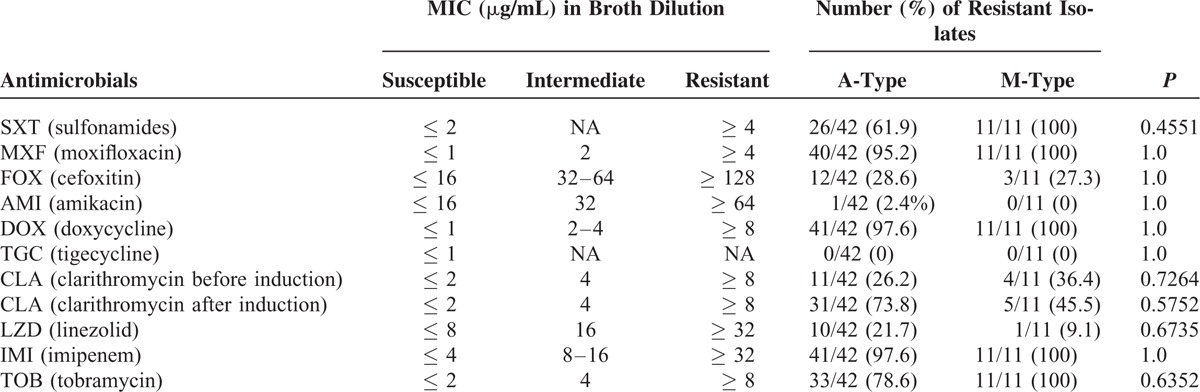
Analysis of Antibiotic Sensitivity of *M. abscessus* (A-) and *M. massiliense* (M-)Type Strains

### MLST Sequencing and Analysis of STs

**FIGURE 1:**
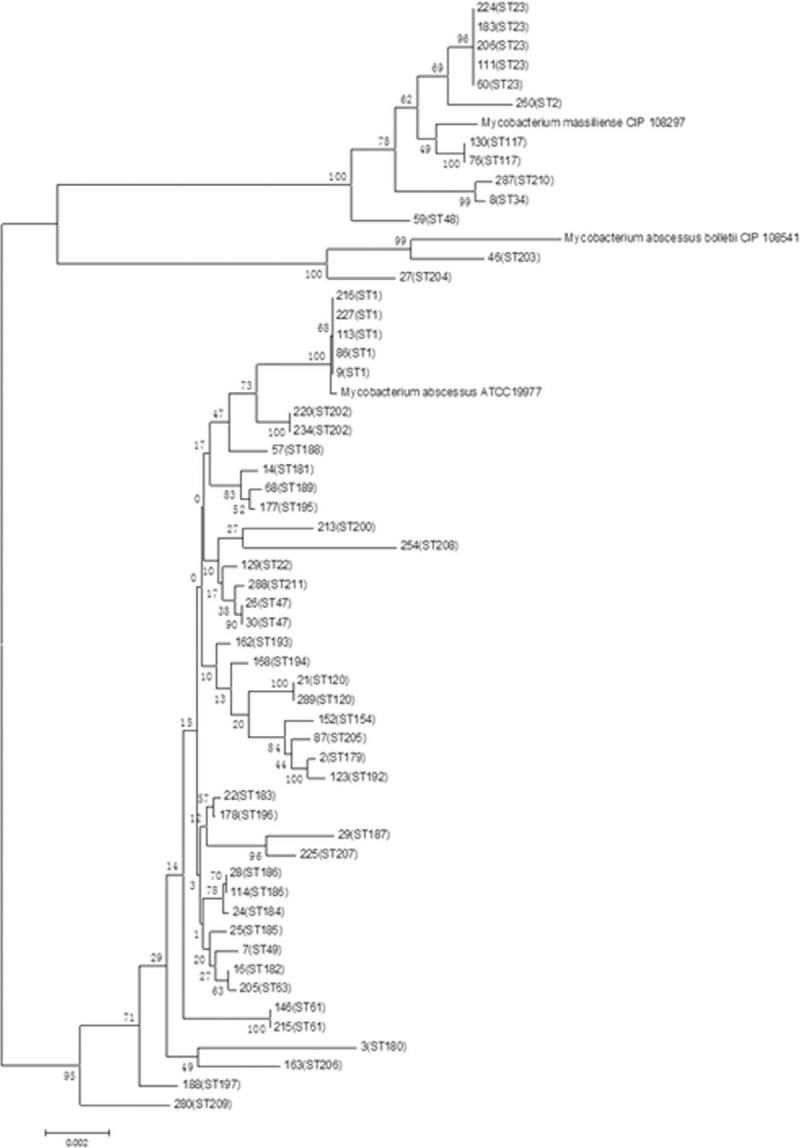
Distribution of sequence types (STs) in 55 isolates by multilocus sequence typing (MLST). MLST = multilocus sequence typing, ST = sequence types.

A comparative analysis utilizing references in the website www.pasteur.fr/mlst found neither base loss nor insertion in all the 7 housekeeping genes examined. Additionally, a new gene, ArgH34, was identified. Analysis of the 55 isolates demonstrated that the sequence types ST1 in the A-type and ST 23 in the M-type were in the majority and that 32 new STs were identified. The data was submitted to the allele distribution inquiry website and new numbers ST179-ST211 were assigned. Bacteria isolates were subjected to the aggregation analysis by the NJ method together with reference strains (ATCC19977 for A-type, CIP18297 for M-type, and CIP108541 for B-type) and categorized into 3 groups; 42 strains in A-type with 33 STs, 2 in B-type with 2 STs, and 11 in M-type with 6 STs (Figure 1). The result was consistent with the sequencing analysis of the rpoB, erm(41), and PRA-hsp65 genes.

### Phylogenetic Relationship Analysis

The analysis of the phylogenetic relationship between *M. abscessus* isolates was carried out using the MST method and utilizing the MLST sequencing results. Known strains containing 178 STs, published on the website www.pasteur.fr/mlst, were used as a reference. The 55 isolated were distributed to 12 CC out of 18 (we downloaded the data that was published in the Pasteur website and then used Bionumber software for re-analysis) that consisted of altogether 178 STs. A-type strains were assigned to 8 CC (CC4-CC11), M-type to 3 CC (CC1-CC3), and B-type to 1 CC (CC12) (Table 3). The maximum clonal complex in the A-type was CC5 consisting of 19 STs. Five A-type strains with 4 different STs (ST120, ST208, ST182, and ST63) were in CC5 and ST63 appeared to be the ancestor of CC5. Another 5 A-type strains with ST1 were assigned to CC11, to which the reference strain *M. abscessus* ATCC19977 was assigned as well. Other A-type strains were assigned to CC6, CC7, and CC8, which were composed of newly identified STs (Table 3). The maximum clonal complex for the M-type strain was CC2 that included 6 strains with 2 different STs and among the 5 strains with ST23 appeared to be the ancestor of CC2. However, the reference strain *M. massiliense* (CIP108297) was not assigned to CC2, rather to other CC in which no strains isolated in our study were placed. Finally, only 1 of 2 B-type strains with ST204 was assigned to CC12.

**TABLE 3:**
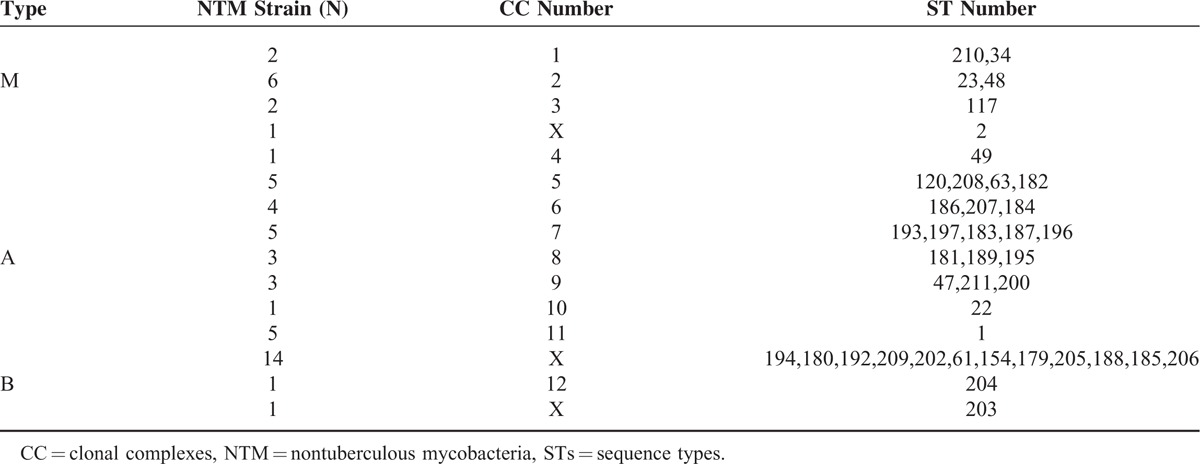
Grouping of *M. massiliense* (M-), *M. abscessus* (A-), and *M. bolletii* (B-)Type Strains According to Clonal Complexes (CC) and Sequence Types (STs)

The NJ method was used to analyze MLST sequencing results with bootstrap confidence interval set at 2000. The maximum likelihood method was used to calculate the phylogenetic relationship, and Mega 6.06 software was used for the analysis. The reference strains used were ATCC19977, CIP18297, and CIP108541.

CC was created by clustering STs according to MLST sequencing results. Each CC was composed of 2 or more STs and each ST contained 6 of the 7 housekeeping genes. The phylogenetic relationship was analyzed by the MST method.

### Phylogenetic Analysis of STs and CC by PFGE

**FIGURE 2:**
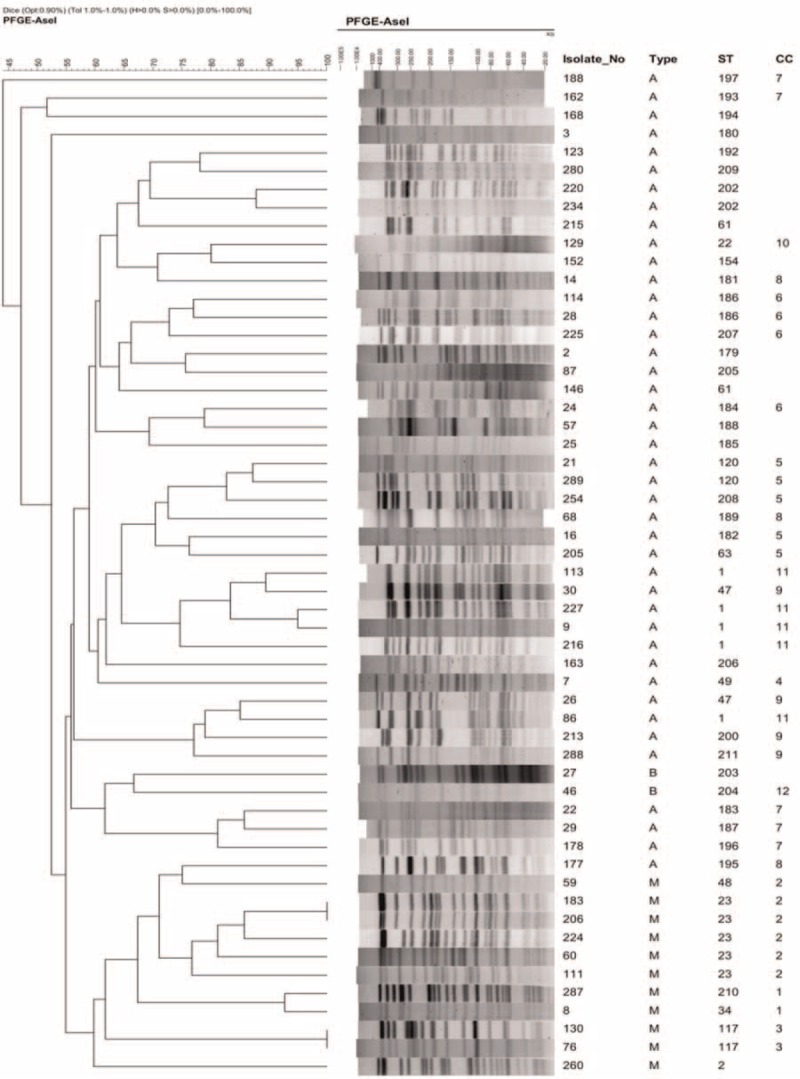
Image of pulsed-field gel electrophoresis (PFGE) restriction patterns of 55 isolates and phylogenetic comparison of sequence types (STs) and clonal complexes (CC). CC = clonal complexes, PFGE = pulsed-field gel electrophoresis, ST = sequence types.

**FIGURE 3:**
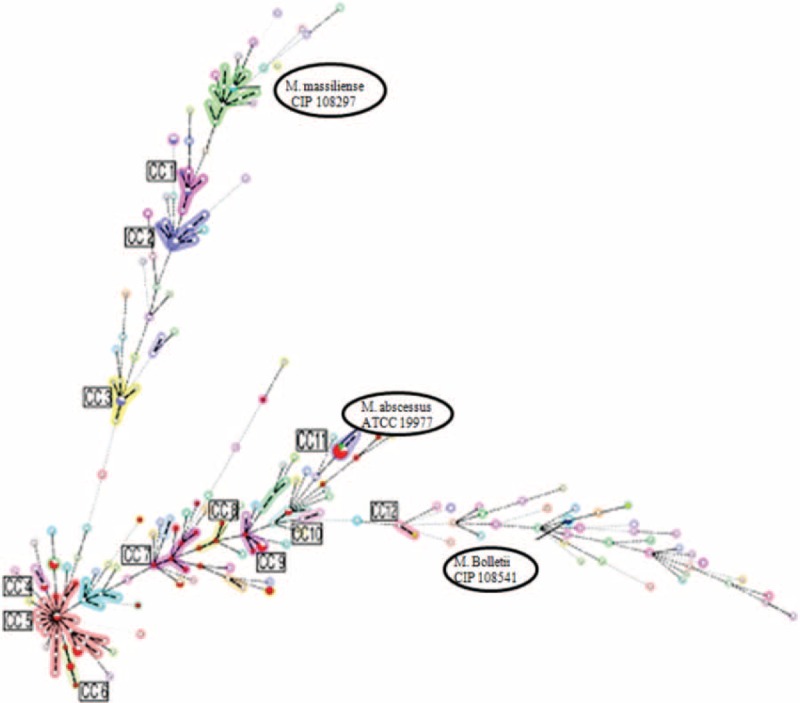
Phylogenic distribution of clonal complexes (CC) in 55 isolates by mining spanning tree (MST). CC = clonal complexes, MLST = multilocus sequence typing.

PFGE analysis of 55 isolates showed 53 distinct restriction patterns, with identical patterns being observed between isolates No. 183 and No. 206, and isolates between No. 130 and No. 76 (Figure 2). Clustering strains according to their PFGE patterns showed that 11 M-type strains were clustered as a big complex and 2 B-type strains were placed side by side among A-type strains. Most of the A-type strains were also clustered; however, isolate No. 177 was clustered with the M-type strains (Figure 3). The PFGE pattern clustering also demonstrated that almost all strains with the same ST numbers clustered together, with the exception of 2 strains with ST47 and ST61. With respect to CC, all M-type strains clustered according to their CC classification, whereas A-type strains assigned to CC6, CC7, CC8, and CC9 were distributed in different clusters, suggesting the possibility that A-type strains belonging to these CC are the new epidemic CC.

Bacteria isolates were subjected to PFGE. The gel images were obtained and analyzed using BioNumerics ver. 5.1. Bacterial genotype grouping and classification, as well as genotype similarity, were obtained utilizing the calculation of the unweighted pair group method with the arithmetic mean (UPGMA).

### Clinical Manifestations and Analysis of CT Results

Because of the limited numbers of STs and CC in isolated strains in this study, it was difficult to analyze the relationships between molecular typing, drug sensitivity, and clinical manifestations. Therefore, clinical characteristics and CT images of 55 patients were analyzed according to the subtypes of *M. abscessus.* Among 42 patients infected with the A-type strain, 22 were men and 20 were women, with a median age of 57 years. Among 11 patients infected with the M-type strain, 5 were men and 6 were women, and the median age was 52 years. No statistically significant differences were found in the distribution of gender, age, smoking history, underlying diseases, or clinical symptoms in these 2 groups. The patients’ clinical characteristics are summarized in Table 4.

**TABLE 4:**
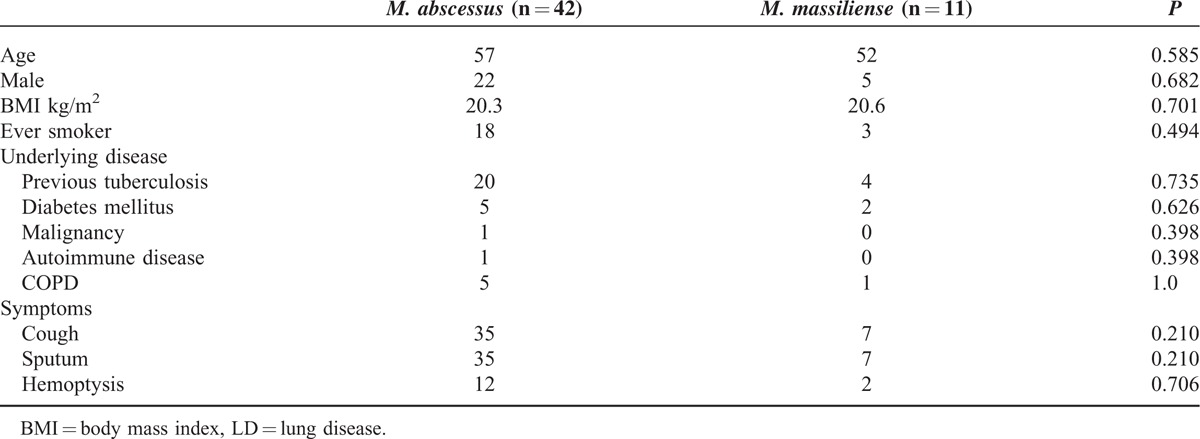
Clinical Characteristics of Patients With *M. abscessus*-LD and *M. massiliense*-LD

**FIGURE 4:**
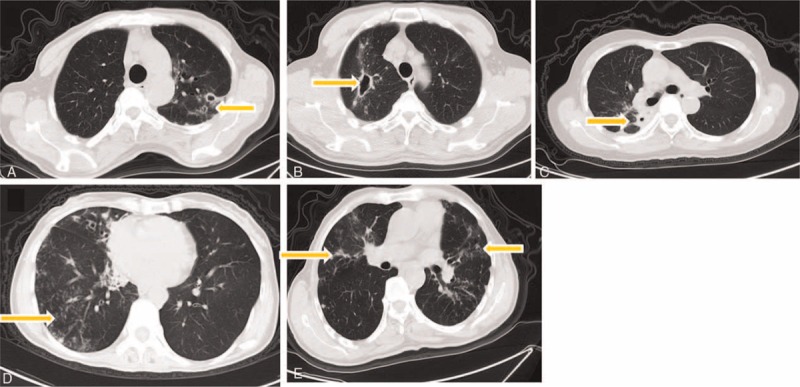
CT images of patients infected with *M. abscessus* (A-type) and *M. massiliense* (M-type). (A) Thin-walled small cavity in A-type patients’ CT scan, (B) thin-walled big cavity in A-type patients’ CT scan, (C) thick-walled small cavity in A-type patients’ CT scan, (D) tree-in-bud pattern in M-type patients’ CT scan, (E) patchy in M-type patients’ CT scan.

CT manifestations of patients with A- and M-type bacterial infection are summarized in Table 5. Common CT manifestations observed in A-type patients were bronchiectasis (39/42), cavity of various types (29/42, 17/42, 25/42 and 9/42), and patch shadow (13/42), whereas the CT manifestations in M-type patients were bronchiectasis (8/11), patch shadow (4/11), and tree-in-bud (5/11). Manifestations such as thin-walled small cavity (*d* < 3 cm) (*P* = 0.002), thin-walled big cavity (*d* ≥ 3 cm) (*P* = 0.005), and thick-walled small cavity (*d* < 3 cm) (*P* = 0.001) (Figure 4A–C) were more common in A-type patients (Table 6). CT examination further showed that in patients with thin-walled small cavities (*d* < 3 cm) and patients with thick-walled small cavities, the range of the lesion involving the lung lobe was more extensive. CT manifestations such as tree-in-bud and patch shadow (Figure 4D and E) were more common in M-type patients (*P* < 0.001, *P* = 0.012). The incidence rate of overall cavities in A-type patients was significantly higher than that in M-type patients (*P* < 0.001) (Table 6).

**TABLE 5:**
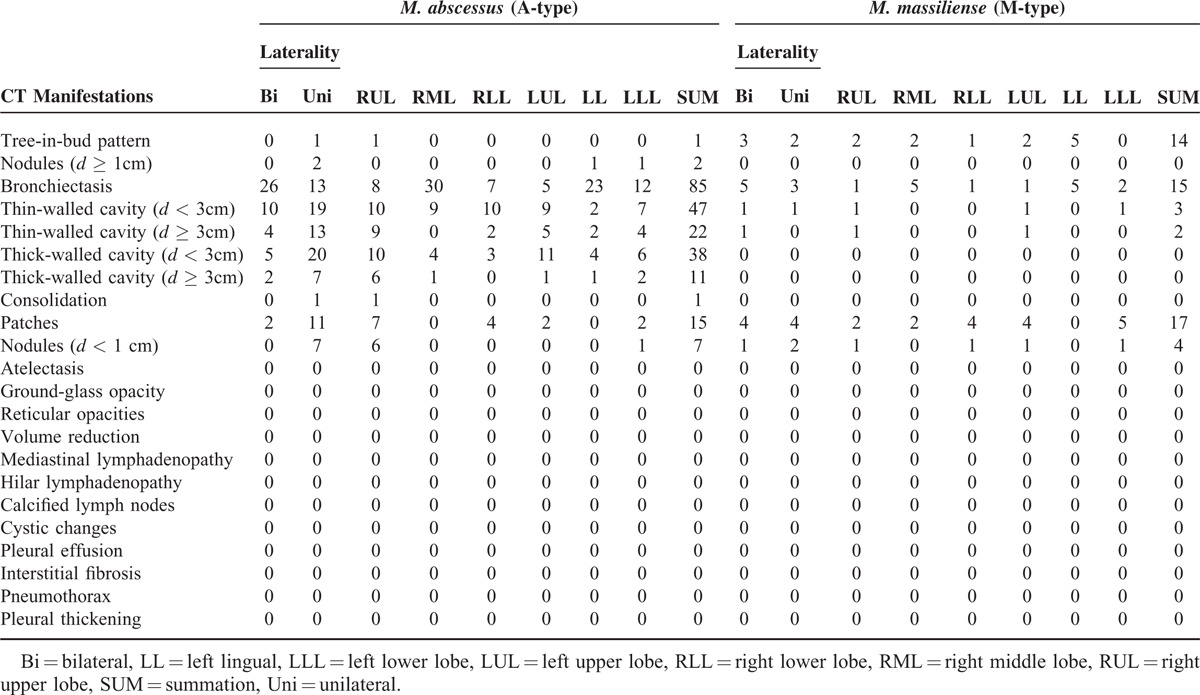
Laterality and Distribution of Parenchymal Lesions in Patients With *M. abscessus* (A-Type) and *M. massiliense* (M-Type)

Multislice spiral CT scanning was performed with 5 mm thick images at 5 mm intervals: A: thin-walled small cavity (*d* < 3 cm); B: thin-walled big cavity (*d* ≥ 3 cm); C: thick-walled small cavity (*d* < 3 cm); D: tree-in-bud; E: patch shadow.

## DISCUSSION

Our study showed that the A-subtype of *M. abscessus* is probably a major epidemic NTM in the Shanghai District of China, considering the high infection rate (76.4%, 42 of 55 patients) found in the present study. The result was consistent with findings in Beijing, Guangzhou, and other cities in China and in Europe as well; however, Korean research has shown that 46.8% (22 of 47 patients) had a slightly lower infection rate of the A-type.^9^ The drug sensitivity test showed that both the A-type and M-type were highly sensitive to tigecycline (A-type 100% and M-type 100%) and amikacin (A-type 97.6% and M-type 100%), in agreement with the results reported by Jeon et al (2014).^9^ The later authors tested the same 10 antibiotics except tigecycline and found that *M. abscessus* subtypes were highly sensitive to amikacin (A-type 96.2% and M-type 90.9%). Lyu et al (2014)^20^ also reported that amikacin was effective against both the A-type and M-type, and that clarithromycin had superior efficacy to amikacin. In our study, cefoxitin, clarithromycin, and linezolid showed some effectiveness against these 2 subtypes; however, the drug resistance to clarithromycin was easily induced in the A-type but not in the B-type (Table 2). *Mycobacterium abscessus* was highly resistant to moxifloxacin, doxycycline, imipenem, and tobramycin. Although the M-type is reported to be more resistant to imipenem than the A-type,^17^ we found that both subtypes were similarly highly resistant to imipenem (100% and 97.6%). Therefore, we recommend tigecycline and amikacin as the preferred treatment for *M. abscessus* pulmonary diseases in the clinic. Clarithromycin appeared to be more suitable to treat the M-type rather than the A-type, because of its lower rate of induced drug resistance.

We performed molecular typing of 55 bacteria strains isolated from patients and analyzing them with PFGE, MLST, and MST. A total of 32 new STs (ST179-ST211) were identified by MLST sequencing. Among all the detected STs, ST1 in A-type and ST23 in M-type were the major sequence types, strongly suggesting that they are predominantly responsible for epidemic transmission in the Shanghai District. This result agrees well with findings in other countries and regions in Europe, America, and Asia, substantiating the speculation that ST1 and ST23 are probably global epidemic types.^21^ The MST method was used to analyze further the phylogenic relationships of the 55 isolates together with the reference strains with 178 STs. STs identified in our isolates were distributed in 12 different CC, and 5 of the A-type strains with ST1 were allocated in CC11, 5 with 4 different STs in CC5, and ST63 was speculated to be the ancestor of CC5, which is consistent with the European study,^22^ which reported 11 STs newly identified in A-type strains, and among those ST186, ST196, and T189 appeared to be the ancestors of these CC, respectively. This result could enrich the database of the A-type strain and also suggests that some of the newly created CC might be specific to and have originated in the Shanghai District of China; however, further studies are required to back up this supposition. In M-type strains, ST23 was most abundant followed by ST117 and ST34. All of them finely aggregated into clusters with STs identified in Brazil, Ireland, France, and other European countries.^23^

To further assess the epidemic dissemination of these strains, we carried out PFGE typing analysis. The 55 isolates showed 53 distinct restriction patterns. The PFGE pattern showed that M-type strains clustered together according to their ST and CC numbers, consistent with the MLST analysis (Figure 2). Since Bryant et al (2013)^22^ reported that M-type strains with small sequence variations could indicate epidemical nosocomial transmission among patients, the M-type might be largely responsible for such transmission in the Shanghai district as well. Although the A-type was the most common bacteria in our isolates, they, except one with ST1, dispersed into different clusters in the PFGE analysis. The results suggested that M-type strains also play an important role in epidemic transmission in this region; therefore, an expanded study with larger numbers of M-type strains is currently in progress and we plan to perform next-generation sequencing to receive more detailed information, such as SNPs and more MLST genes for comparison, which might be helpful for us to identify transmission routes.

Clinical treatment is a complicated and a time-consuming process. NTM pulmonary diseases are extremely difficult to treat, as shown by Japanese research in which the antibiotic treatment of patients infected with A- subtypes of *M. abscessus* required a longer mean duration of treatment (3–178 months) compared to M-subtypes (1–122 months). The Japanese research also reported that compared to patients with M-type, patients with A-type showed less improvement after antibiotic treatment, as evidenced by CT imaging. We found no significant difference between patients with the A-type and B-type in their clinical baseline characteristics, such as age and gender (Table 4). However, CT imaging analysis showed that in patients infected with the A-type, the rate of occurrence of pulmonary cavities was significantly higher than in patients with M-type. Furthermore, these cavities were characterized mainly by thin-walled small cavities and thick-walled small cavities, with more extensive lung lobe involvement (Tables 5 and 6). Harada et al reported that their antibiotic sensitivity tests, clinical treatments of patients, and follow-up observations revealed that drug treatments are more effective in patients with M-type than A-type infections, although bronchiectasis was more common in A-type (*P* = 0.014).^17^ This finding was supported by Koh et al^18^ who reported that the A-type strain was more prone to drug resistance resulting in a poor response to antibiotics such as clarithromycin. Our finding of frequent and severe pulmonary cavities in patients with the A-type strain is in good agreement with the later report. Formation of cavities makes infection more refractory, reducing the therapeutic effect, delaying healing and also increasing the risk of the induction of drug resistance. In addition, pulmonary imaging of patients with A-type infections showed small airway inflammation and inflammatory exudation. The extent of pulmonary destruction in patients with the M-type was less severe and the prognosis of these patients was superior to that of A-type patients. However, it is worth noting that both A- and M-type strains, without statistical differences, cause a wide range of bronchiectasis with different degrees of nodule shadows.

In conclusion, we found that ST1 in the A-subtype and ST23 in the M-subtype of *M. abscessus* isolated in the Shanghai district of China were the main sequence types of epidemic strains. The M-type strain was more prone to epidemic nosocomial transmission. Clinical treatment of A-type patients with clarithromycin needs to be carefully monitored because of the frequent occurrence of drug resistance. A combination treatment of tigecycline and amikacin is recommended and if appropriate the use of a combination of linezolid and cefoxitin should be considered as an alternative therapeutic strategy.

## References

[1] Brown-Elliott BA Wallace RJ Jr Clinical and taxonomic status of pathogenic nonpigmented or late-pigmenting rapidly growing mycobacteria. *Clin Microbiol Rev* 2002; 15:716–746. 12364376 10.1128/CMR.15.4.716-746.2002 PMC126856

[2] Tiwari TS Ray B Jost KC Jr Forty years of disinfectant failure: outbreak of postinjection *Mycobacterium abscessus* infection caused by contamination of benzalkonium chloride. *Clin Infect Dis* 2003; 36:954–962. 12684906 10.1086/368192

[3] Villanueva A Calderon RV Vargas BA Report on an outbreak of postinjection abscesses due to *Mycobacterium abscessus*, including management with surgery and clarithromycin therapy and comparison of strains by random amplified polymorphic DNA polymerase chain reaction. *Clin Infect Dis* 1997; 24:1147–1153. 9195073 10.1086/513656

[4] Wallace RJ Jr Brown BA Griffith DE Nosocomial outbreaks/pseudo-outbreaks caused by nontuberculous mycobacteria. *Annu Rev Microbiol* 1998; 52:453–490. 9891805 10.1146/annurev.micro.52.1.453

[5] Tortoli E Clinical manifestations of nontuberculous mycobacteria infections. *Clin Microbiol Infect* 2009; 15:906–910. 19845702 10.1111/j.1469-0691.2009.03014.x

[6] Griffith DE Aksamit T Brown-Elliott BA An official ATS/IDSA statement: diagnosis, treatment, and prevention of nontuberculous mycobacterial diseases. *Am J Respir Crit Care Med* 2007; 175:367–416. 17277290 10.1164/rccm.200604-571ST

[7] Benwill JL Wallace RJ Jr *Mycobacterium abscessus*: challenges in diagnosis and treatment. *Curr Opin Infect Dis* 2014; 27:506–510. 25268925 10.1097/QCO.0000000000000104

[8] Chadha R Grover M Sharma A An outbreak of post-surgical wound infections due to *Mycobacterium abscessus*. *Pediatr Surg Int* 1998; 13:406–410. 9639628 10.1007/s003830050350

[9] Jeon SM Lim NR Kwon SJ Analysis of species and intra-species associations between the *Mycobacterium abscessus* complex strains using pulsed-field gel electrophoresis (PFGE) and multi-locus sequence typing (MLST). *J Microbiol Methods* 2014; 104:19–25. 24918987 10.1016/j.mimet.2014.05.024

[10] O’Driscoll C Konjek J Heym B Molecular epidemiology of *Mycobacterium abscessus* complex isolates in Ireland. *J Cyst Fibros* 2015; DOI: 10.1016/j.jcf.2015.05.007. 10.1016/j.jcf.2015.05.007 26072272

[11] Society, AT. Diagnosis and treatment of disease caused by nontuberculous mycobacteria. This official statement of the American Thoracic Society was approved by the Board of Directors, March 1997. Medical Section of the American Lung Association. *Am J Respir Crit Care Med* 1997; 156:S1–25. 9279284 10.1164/ajrccm.156.2.atsstatement

[12] ANVIS.A. Relatóriodescrito de investigação de casos de infecçõespormicobactériasnão tuberculosis de crescimentorápido (MCR) no Brasilnoperíodo de 1998 a 2009. *Agência Nacional de VigilânciaSanitária, Brasilia, Brazil* 2012; http://www.anvisa.gov.br/hotsite/hotsite_micobacteria/relatorio_descrito_mcr_16_02_11.pdf.

[13] Adekambi T Colson P Drancourt M rpoB-based identification of nonpigmented and late-pigmenting rapidly growing mycobacteria. *J Clin Microbiol* 2003; 41:5699–5708. 14662964 10.1128/JCM.41.12.5699-5708.2003 PMC308974

[14] Leao SC Tortoli E Euzeby JP Proposal that *Mycobacterium massiliense* and *Mycobacterium bolletii* be united and reclassified as *Mycobacterium abscessus* subsp. bolletii comb. nov., designation of *Mycobacterium abscessus* subsp. abscessus subsp. nov. and emended description of *Mycobacterium abscessus*. *Int J Syst Evol Microbiol* 2011; 61:2311–2313. 21037035 10.1099/ijs.0.023770-0

[15] Telenti A Marchesi F Balz M Rapid identification of mycobacteria to the species level by polymerase chain reaction and restriction enzyme analysis. *J Clin Microbiol* 1993; 31:175–178. 8381805 10.1128/jcm.31.2.175-178.1993 PMC262730

[16] Diagnosis and treatment of disease caused by nontuberculous mycobacteria. This official statement of the American Thoracic Society was approved by the Board of Directors, March 1997. Medical Section of the American Lung Association. *Am J Respir Crit Care Med* 1997; 156:S1–S25.http://www.atsjournals.org/doi/abs/10.1164/ajrccm.156.2.atsstatement?url_ver=Z39.88-2003&rfr_id=ori%3Arid%3Acrossref.org&rfr_dat=cr_pub%3Dpubmed&#.Vo8cldJAXX4. 9279284 10.1164/ajrccm.156.2.atsstatement

[17] Harada T Akiyama Y Kurashima A Clinical and microbiological differences between *Mycobacterium abscessus* and *Mycobacterium massiliense* lung diseases. *J Clin Microbiol* 2012; 50:3556–3561. 22915613 10.1128/JCM.01175-12 PMC3486228

[18] Koh WJ Jeon K Lee NY Clinical significance of differentiation of *Mycobacterium massiliense* from *Mycobacterium abscessus*. *Am J Respir Crit Care Med* 2011; 183:405–410. 20833823 10.1164/rccm.201003-0395OC

[19] Clinical and Laboratory Standards Institute. Susceptibility testing of mycobacteria, nocardia, and other aerobic actinomycetes; approved Standard M24-A2. Wayne, PA: CLSI; 2011. 31339680

[20] Lyu J Kim BJ Kim BJ A shorter treatment duration may be sufficient for patients with *Mycobacterium massiliense* lung disease than with *Mycobacterium abscessus* lung disease. *Respir Med* 2014; 108:1706–1712. 25245792 10.1016/j.rmed.2014.09.002

[21] Choo SW Wee WY Ngeow YF Genomic reconnaissance of clinical isolates of emerging human pathogen *Mycobacterium abscessus* reveals high evolutionary potential. *Sci Rep* 2014; 4:4061. 24515248 10.1038/srep04061 PMC3920477

[22] Bryant JM Grogono DM Greaves D Whole-genome sequencing to identify transmission of *Mycobacterium abscessus* between patients with cystic fibrosis: a retrospective cohort study. *Lancet* 2013; 381:1551–1560. 23541540 10.1016/S0140-6736(13)60632-7 PMC3664974

[23] Davidson RM Hasan NA de Moura VC Phylogenomics of Brazilian epidemic isolates of *Mycobacterium abscessus* subsp. bolletii reveals relationships of global outbreak strains. *Infect Genet Evol* 2013; 20:292–297. 24055961 10.1016/j.meegid.2013.09.012 PMC3853122

